# Serum asunaprevir concentrations showing correlation with the extent of liver fibrosis as a factor inducing liver injuries in patients with genotype-1b hepatitis C virus receiving daclatasvir plus asunaprevir therapy

**DOI:** 10.1371/journal.pone.0205600

**Published:** 2018-10-11

**Authors:** Yoshihito Uchida, Kayoko Naiki, Jun-ichi Kouyama, Kayoko Sugawara, Masamitsu Nakao, Daisuke Motoya, Mie Inao, Nobuaki Nakayama, Yukinori Imai, Tomoaki Tomiya, Satoshi Mochida

**Affiliations:** Department of Gastroenterology & Hepatology, Faculty of Medicine, Saitama Medical University, Moroyama, Saitama, Japan; Nihon University School of Medicine, JAPAN

## Abstract

**Aims:**

Liver injury can occur during antiviral therapies with direct-acting antivirals (DAAs), potentially necessitating discontinuation of the therapies, with consequent worsening of the sustained viral response (SVR) rates, in patients with hepatitis C virus (HCV). To clarify the mechanisms involved in serum transaminase level elevation, we performed a retrospective evaluation of the serum concentrations of daclatasvir and asunaprevir, both classified as DAAs, in patients receiving treatment with a combination of the two drugs.

**Methods:**

Subjects were 278 Japanese patients with genotype-1b HCV who received daclatasvir plus asunaprevir therapy for more than 4 weeks. Serum concentrations of both the DAAs were measured at 4 weeks after the initiation of therapy.

**Result:**

Liver injuries including serum AST and/or ALT level elevation to 150 U/L or over were found in 34 patients (12.2%). Multivariate logistic regression analysis identified serum asunaprevir concentrations as being significantly associated with developing liver injury, with an odds ratio of 1.046 (95% confidence interval 1.011–1.082, *p<*0.05). Serum asunaprevir concentrations showed correlation with the extent of liver fibrosis, estimated by peripheral platelets counts and serum albumin levels and baseline and FIB4 index and serum Mac-2 binding protein glycosylation isomer (M2BPGi) levels at 4 weeks of the therapy; the concentrations were significantly higher among patients showing 3.0 or more of M2BPGi levels than among those with the levels less than 3.0; on the other hand, no such correlation/difference was found in serum daclatasvir concentrations.

**Conclusion:**

High serum concentrations of serum asunaprevir, which were associated with the extent of liver fibrosis, appear to provoke the occurrence of liver injury in patients with genotype-1b HCV receiving combined daclatasvir plus asunaprevir therapy.

## Introduction

Treatment using direct-acting antivirals (DAAs) with or without ribavirin (RBV) has resulted in markedly improved sustained viral response (SVR) rates in patients with hepatitis C virus (HCV). In general, 3 types of DAAs have been used in patients with HCV: NS3/4A protease inhibitors, NS5A replication complex inhibitors, and nucleotide/non-nucleotide NS5B polymerase inhibitors. In Japan, dual oral therapy with daclatasvir (DCV), an NS5A replication complex inhibitor, and asunaprevir (ASV), a second-generation NS3/4A protease inhibitor, was approved in July 2014 as the first interferon-free treatment regimen for patients with genotype 1b HCV. In patients receiving DCV plus ASV therapy, presence of resistance-associated substitutions (RASs), especially Y93H and L31M/V substitutions in NS5A region, at the baseline was shown to be associated with virologic failure [1]. Thus, we developed the diagnostic system to evaluate the presence/absence of NS5A-RASs [2, 3]. Although the pre-treatment examination of NS5A-RASs had contributed to an improvement in the therapeutic efficacy of DCV plus ASV therapy in real-world clinical practice, deterioration of the therapeutic efficacy of DCV plus ASV therapy had been reported in patients in whom the therapy had to be discontinued due to the appearance of adverse events [4].

Among the adverse events associated with DCV plus ASV therapy, elevation of the serum transaminase and bilirubin levels often necessitates discontinuation of the treatment [1, 4]. It is considered that ASV, but not DCV, is responsible for the liver injury in patients receiving this combination treatment, because abnormal liver test results were frequently encountered during triple therapy with ASV, RBV and pegylated interferon (Peg-IFN) [5], but were infrequent during therapy with DCV, RBV and Peg-IFN [6]. Thus, in the present study, we retrospectively evaluated the serum concentrations of DAAs in patients receiving DCV plus ASV therapy and assessed the significance of the drug levels in relation to elevation of the serum aspartate aminotransferase (AST) and/or alanine aminotransferase (ALT) levels during the therapy, in order to clarify the mechanisms involved in the occurrence of liver injury as an adverse event in patients receiving this treatment.

## Patients and methods

### Patients and study design

The present retrospective study was subjecting the patients who enrolled in our previous clinical observational study. Namely, the subjects were 278 Japanese patients with genotype 1b HCV who were enrolled in the previous clinical observational study for treatment with DCV plus ASV therapy for 24 weeks between September 2014 and November 2015 at Saitama Medical University Hospital (Moroyama, Saitama, Japan). All the patients were administered DCV (Daklinza; Bristol-Myers Squibb, NY, USA) at the dose of 60 mg once a day, and ASV (Sunvepra; Bristol-Myers Squibb) at the dose of 100 mg twice a day without discontinuation of the therapy within 4 weeks. Serum samples were collected at baseline and at 4 weeks after the initiation of therapy. The former samples were subjected to evaluation of NS5A-RASs of HCV strains and the extent of liver fibrosis, while the latter samples for measurement of DCV and ASV concentrations as well as estimation of liver fibrosis staging.

The study conformed to the ethical guidelines of the Declaration of Helsinki and was conducted with the approval of the Institutional Review Board of Saitama Medical University Hospital as 16-053-2 and 15–073, and the registration to the University Hospital Medical Information Network Clinical Trials Registry (UMIN-CTR) as UMIN000018857. Written informed consent including possible further studies was obtained from all the patients prior to the previous clinical study for DCV plus ASV therapy (15–073 and UMIN000018857), and informed consent for the present retrospective study (16-053-2) was obtained in the form of opt-out consent on the website of Saitama Medical University Hospital (http://www.saitama-med.ac.jp/hospital/outline/irb_kouhou.html) from all of the patients. The patients who rejected to the study were excluded.

### Determination of NS5A-RASs of HCV strains and the extent of liver fibrosis and injuries

The percentages of HCV strains with NS5A-Y93H substitutions to total HCV-RNA titers were evaluated by cycling-probe real-time PCR assay [2], and the amino acid sequences of NS5A regions were analyzed by direct sequencing according to previously reported methods [2, 3].

The extent of liver fibrosis estimated from peripheral platelet count, FIB4 index and serum Mac-2 binding protein glycosylation isomer (M2BPGi) level [7, 8]. Liver injuries were defined as elevation of the serum AST and/or ALT levels to 150 U/L or more, and the appearance of ascites, hepatic edema, hyperammonemia and hepatic encephalopathy during DCV plus ASV therapy.

### Measurement of the serum concentrations of daclatasvir and asunaprevir

Serum concentrations of DCV and ASV were determined using validated liquid chromatography with tandem mass spectrometry methods using serum samples frozen at -140°C by CIMIC Pharma Science Co., Ltd. (Tokyo, Japan). [9, 10].

### Statistical analysis

Categorical data was compared using Fisher’s exact test. Distributions of continuous variables were analyzed using Mann–Whitney *U*-test. Multiple logistic regression analysis was conducted to identify factors associated with liver injury. The correlation coefficient (r) was obtained by calculation of the Spearman’s rank correlation coefficient. All tests of significance were two-tailed, and *p*<0.05 was considered as denoting statistical significance. SPSS Statistics version 22 (IBM SPSS, Tokyo, Japan) was used for the analysis.

## Results

### Demographic and clinical features of patients receiving daclatasvir plus asunaprevir therapy

A total of 278 patients, consisting of 129 men and 149 women, with a median age of 70 years (range, 23 to 88 years), received DCV plus ASV therapy. This cohort of 278 patients contained the 206 patients included in a previous study that showed the existence of a correlation between the therapeutic efficacy of DAAs and the presence of NS5A-RASs at the baseline [4]. Of the 278 patients, 64 (23.0%) had underlying compensated cirrhosis, 39 (14.0%) had received therapies for hepatocellular carcinoma (HCC), and 13 (4.7%) had previous history of antiviral therapies with NS3/4A protease inhibitors such as telaprevir or simeprevir. Furthermore, 246 patients (88.5%) were infected with HCV strains not harboring either NS5A-L31M/V or NS5A-Y93H substitutions, 6 (2.2%) were infected with HCV strains with NS5A-L31M/V substitutions, and 26 (9.4%) were infected with strains with NS5A-Y93H substitutions. The demographic and clinical features of the patients are summarized in Table 1.

**Table 1:** The demographic and clinical features of 278 patients with genotype 1b hepatitis C virus (HCV) infection receiving daclatasvir (DCV) plus asunaprevir (ASV) therapy.

		All patients (n = 278)	Patients without liver injuries (n = 244)	Patients with liver injuries (n = 34)	*p*-value
Age (years old) †		70 (23–88)	70 (23–88)	69 (45–83)	0.806 ^a^
Men / Women††		129 (46.4) / 149 (53.6)	115 (47.1) / 129 (52.9)	14 (41.2) /20 (58.8)	0.584 ^b^
Body weight (Kg) †		55 (30–86)	56 (30–86)	54 (37–78)	0.528 ^a^
Compensated Cirrhosis††		64 (23.0)	53 (21.7)	11 (32.4)	0.192 ^b^
Previous history with administration of NS3/4A protease inhibitors††		13 (4.7)	12 (4.9)	1 (2.9)	1.000 ^b^
Previously treated HCC††		39 (14.0)	34 (13.9)	5 (14.7)	1.000 ^b^
NS5A-L31 substitution††		6 (2.2)	6 (2.5)	0 (0)	1.000 ^b^
NS5A-Y93 substitution††		26 (9.4)	22 (9.0)	4 (11.8)	0.538 ^b^
HCV-RNA (log IU/mL) †		6.2 (2.3–7.3)	6.2 (2.3–7.3)	6.1 (4.5–7.3)	0.561 ^a^
Platelets (10^3^/mm^3^) †	at baseline	140 (30–303)	141 (30–303)	134 (63–264)	0.737 ^a^
	at 4 weeks	152 (30–372)	152 (30–372)	141 (58–303)	0.718 ^a^
Albumin (g/dL) †	at baseline	3.9 (2.3–5.3)	3.9 (2.3–5.3)	3.8 (2.6–4.5)	0.157 ^a^
	at 4 weeks	3.9 (2.4–5.2)	4.0 (2.4–5.2)	3.8 (2.7–4.5)	0.012 ^a^
AST (U/L) †	at baseline	40 (12–218)	39 (11–218)	45 (16–105)	0.405 ^a^
	at 4 weeks	24 (10–1797)	24 (10–75)	28 (15–1797)	0.014 ^a^
ALT (U/L) †	at baseline	31 (5–200)	31 (5–200)	34 (11–111)	0.482 ^a^
	at 4 weeks	16 (6–700)	15 (6–85)	21 (10–700)	0.005 ^a^
Bilirubin (mg/dL) †	at baseline	0.7 (0.3–3.1)	0.6 (0.3–3.1)	0.8 (0.3–2.4)	0.064 ^a^
	at 4 weeks	0.7 (0.1–4.1)	0.7 (0.1–4.1)	0.7 (0.1–1.7)	0.231 ^a^
Creatinine (mg/dL) †	at baseline	0.69 (0.35–13.18)	0.69 (0.35–13.18)	0.69 (0.45–1.81)	0.950 ^a^
	at 4 weeks	0.71 (0.41–12.15)	0.69 (0.35–13.18)	0.69 (0.45–1.81)	0.848 ^a^
eGFR (ml/min/1.73m^2^) †	at baseline	74.0 (3.7–133.2)	74.1 (3.7–133.2)	73.2 (30.6–108.7)	0.788 ^a^
	at 4 weeks	70.1 (4.0–118.0)	70.1 (4.0–118.0)	71.6 (28.5–95.8)	0.716 ^a^
α-fetoprotein (ng/mL) †	at baseline	5.0 (2.0–178.1)	3.8 (2.0–160.6)	5.0 (2.0–178.1)	0.463 ^a^
	at 4 weeks	3.9 (2.0–125.7)	3.9 (2.0–125.7)	3.9 (2.0–46.9)	0.513 ^a^
M2BPGi (C.O.I.)†	at baseline	3.18 (0.37–20.01)	3.10 (0.37–20.01)	3.70 (0.73–20.01)	0.403 ^a^
	at 4 weeks	1.96 (0.31–19.54)	1.91 (0.31–19.54)	2.44 (0.57–16.04)	0.292 ^a^
M2BPGi >3.0 C.O.I.††	at baseline	144 (51.8)	125 (51.2)	19 (55.9)	0.715 ^b^
	at 4 weeks	82 (29.5)	66 (27.0)	16 (47.0)	0.026 ^b^
FIB4 index †	at baseline	3.64 (0.43–19.15)	3.61 (0.43–19.15)	3.79 (0.84–10.08)	0.505 ^a^
	at 4 weeks	2.98 (0.40–62.52)	2.98 (0.40–17.93)	2.68 (0.67–62.52)	0.267 ^a^
FIB4 index >3.25 ††	at baseline	156 (56.1)	136 (55.7)	20 (58.8)	0.854 ^b^
	at 4 weeks	122 (43.9)	106 (43.4)	16 (47.1)	0.715 ^b^
ASV level (ng/dL)†	at 4 weeks	351 (11–6660)	340 (11–6110)	547 (24–6660)	0.085 ^a^
DCV level (ng/dL)†	at 4 weeks	598 (40–2,770)	588 (40–2,770)	732 (142–1,860)	0.260 ^a^

†Medium value (range).

††number of patient (percentages).

^a^ Mann-Whitney *U*-test

^b^ Fisher’s exact test.

*p*-values are for comparison between patients with and without liver injury.

### Outcomes of the dual daclatasvir plus asunaprevir therapy

Among the 278 patients who received DCV plus ASV therapy, the treatment course of 24 weeks was completed in 248 patients (89.2%). Of the remaining 30 patients in whom the treatment was discontinued before the end of 24 weeks, the reason for the treatment discontinuation was virologic failure in 12 patients (4.3%), including 2 and 10 patients with viral rebound and viral breakthrough, respectively, and emergence of adverse events in 18 patients (6.5%). Viral relapse occurred in 14 patients after discontinuation of the DCV plus ASV therapy, including 4 patients in whom the therapy was discontinued due to the appearance of adverse events. Consequently, the results of ITT analysis revealed an overall SVR12 rate of 84.9% (238/278); further analysis revealed an SVR rate of 88.2% (217/246) in patients infected with HCV strains not harboring either NS5A-L31M/V or NS5A-Y93H substitutions, and 100% (6/6) and 57.7% (15/26) in those infected with HCV strains carrying the NS5A-L31M/V substitution and NS5A-Y93H substitution, respectively.

### Serum concentrations of daclatasvir plus asunaprevir

**Fig 1:**
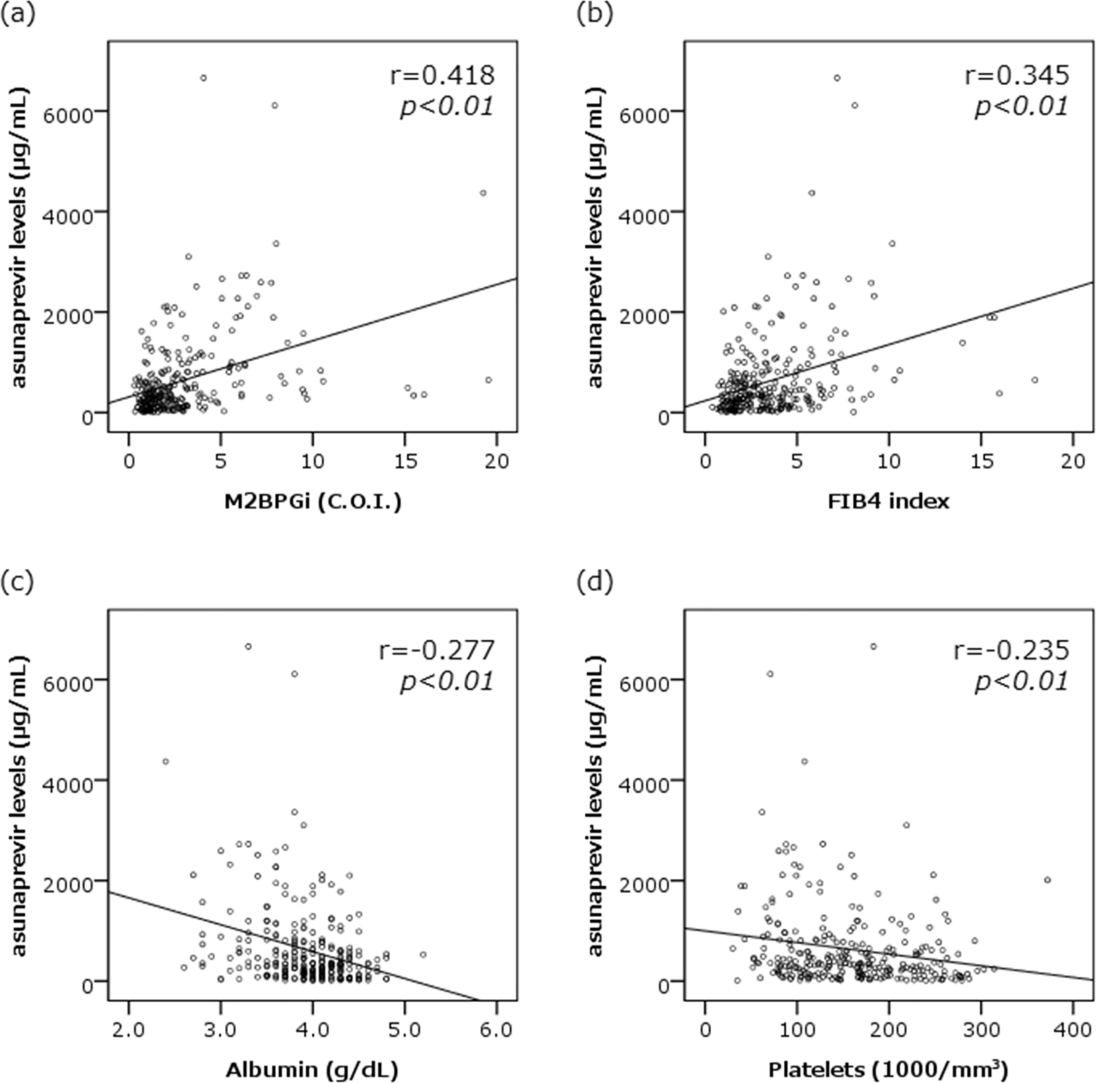
The scatter plots between serum concentrations of asunaprevir and either Mac-2 binding protein glycosylation isomer (M2BPGi) Levels, FIB4 index, serum albumin levels and peripheral platelet counts. Serum asunaprevir concentrations were positively correlated with serum M2BPGi levels (r = 0.418, p<0.001) and FIB4 index (r = 0.345, p<0.001) at 4 weeks after the initiation of DAV plus ASV therapy (a and b). Also, the concentrations were negatively correlated with serum albumin levels (r = -0.277, p<0.001) and platelets counts (r = -0.235, p<0.001) at baseline (c and d).

**Fig 2:**
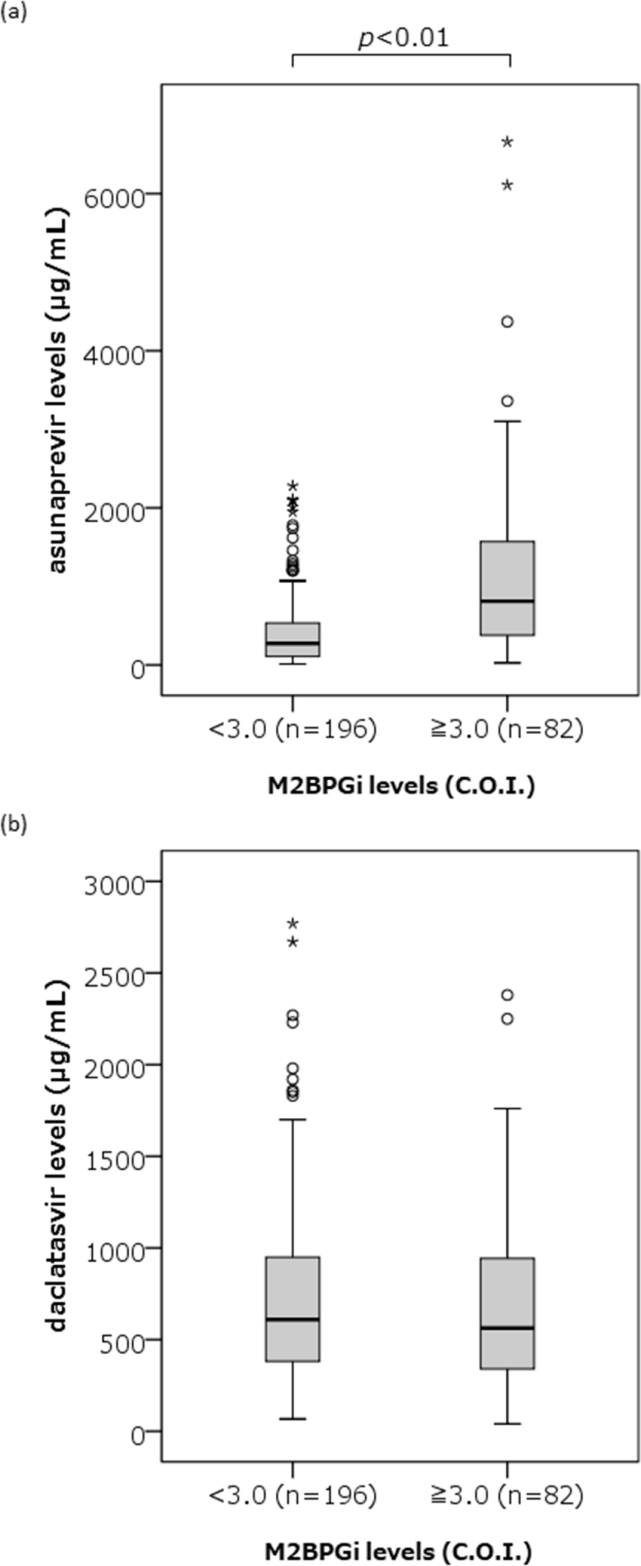
Comparison of serum asunaprevir (ASV) and daclatasvir (DCV) concentrations between patients showing 3.0 C.O.I or more of Mac-2 binding protein glycosylation isomer (M2BPGi) levels and those showing the levels less than 3.0 C.O.I. Serum ASV concentrations were significantly higher in patients showing 3.0 C.O.I or more of M2BPGi levels at 4 weeks than in those with the levels less than 3.0 C.O.I (812; 27–6,600 ng/mL vs. 275; 11–2,280 ng/mL, p < 0.001), but no such difference was observed for the serum DCV concentrations. In these box-and-whisker plots, lines within the boxes represent the median values; the upper and lower lines of the boxes represent the 25th and 75th percentiles, respectively. The tails indicate the minimum and maximum values. Circles indicate outliers of 1.5–3.0 IQR higher than the 75th percentile or 1.5–3.0 IQR lower than the 25th percentile. Asterisks indicate outliers of more than 3.0 IQR higher than the 75th percentile or less than 3.0 IQR lower than the 25th percentile.

**Fig 3:**
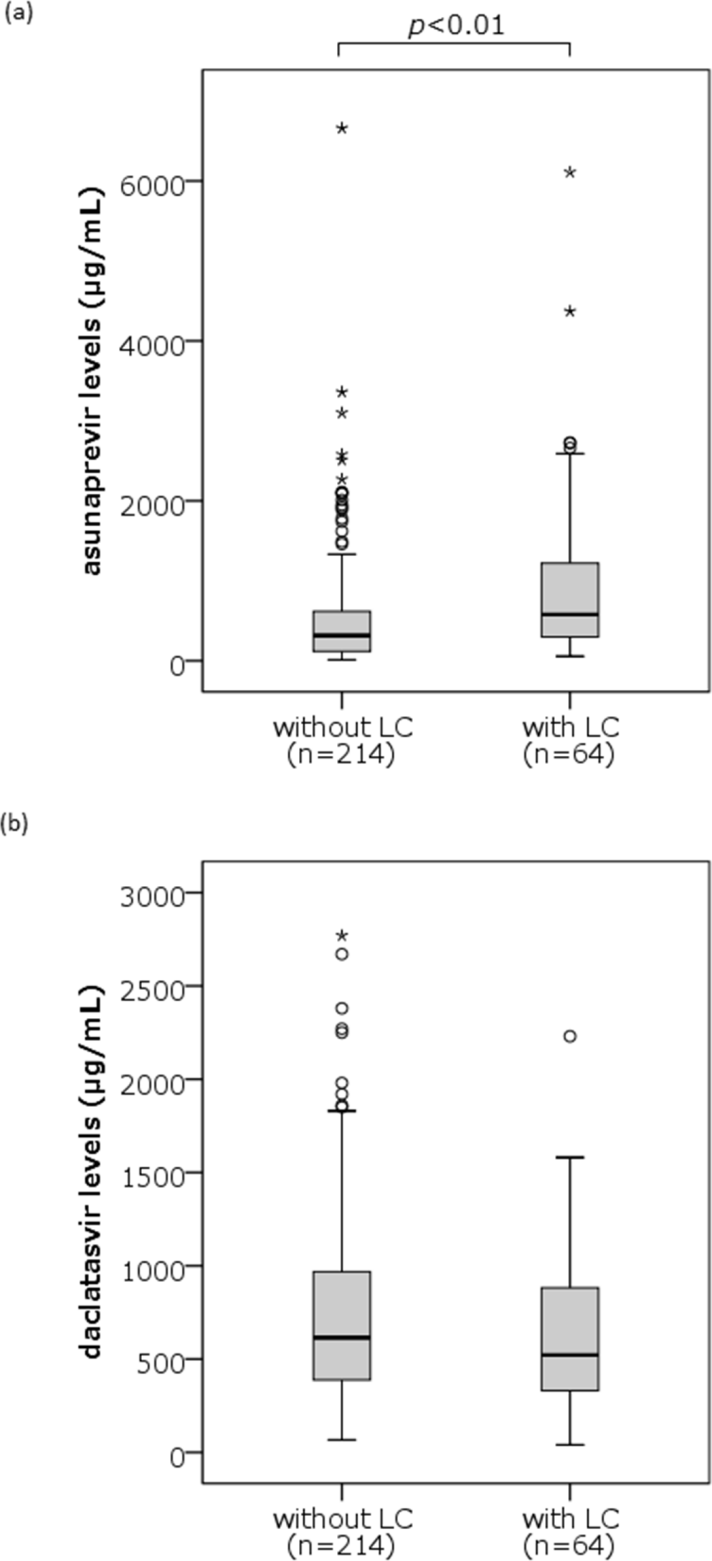
Comparison of serum asunaprevir (ASV) and daclatasvir (DCV) concentrations between patients with liver cirrhosis (LC) and those without LC. Serum ASV concentrations were significantly higher in patients with LC than in those without LC (579; 56–6,110 ng/mL vs. 315; 11–6,660 ng/mL, p < 0.001), but no such difference was observed for the serum DCV concentrations. In these box-and-whisker plots, lines within the boxes represent the median values; the upper and lower lines of the boxes represent the 25th and 75th percentiles, respectively. The tails indicate the minimum and maximum values. Circles indicate outliers of 1.5–3.0 IQR higher than the 75th percentile or 1.5–3.0 IQR lower than the 25th percentile. Asterisks indicate outliers of more than 3.0 IQR higher than the 75th percentile or less than 3.0 IQR lower than the 25th percentile.

Median serum concentrations of ASV and DCV (range) at 4 weeks after the initiation of therapy were 351 (11–6,660) ng/mL and 598 (40–2,770) ng/mL, respectively (***Table 1***). Neither showed any significant correlations with the age, body weight or the following laboratory data at baseline; serum HCV-RNA levels or NS5A-RAS profiles, serum levels of AST, ALT, total bilirubin, creatinine and alpha fetoprotein. Serum ASV concentrations, however, were correlated negatively with peripheral platelet counts and serum albumin levels at baseline, while positively with FIB4 index at 4 weeks after the initiation of therapy. Also, serum ASV concentrations showed the strong correlation with serum M2BPGi levels at 4 weeks of the therapy (r = 0.418, *p*<0.001) (Fig 1). On the other hand, serum DCV concentrations were not correlated with those factors. Furthermore, serum ASV concentrations were also higher in patients showing 3.0 C.O.I or more of M2BPGi levels at 4 weeks (812; 27–6,600 ng/mL) than in those with the levels less than 3.0 C.O.I (275; 11–2,280 ng/mL, *p*<0.001), and higher in patients with liver cirrhosis (579; 56–6,110 ng/mL) than in those with without corrhosis (315; 11–6,660 ng/mL, p<0.001), while no such correlations and differences were observed for the serum DCV concentrations (Figs 2 and 3).

### Factors associated with liver injuries during daclatasvir plus asunaprevir therapy

Liver injuries were observed in 34 patients (12.2%). As shown in ***Table 1***, there were no significant differences in the age, sex or body weight of the patients between those with and without liver injuries. Also, liver cirrhosis, previous antiviral therapies, previous therapies for HCC, the serum HCV-RNA levels and NS5A-RAS profile did not influence on appearance of liver injuries. The serum albumin levels at 4 weeks were significantly lower, and both AST and ALT levels at 4 weeks were significantly higher in patients with liver injuries than in those without liver injuries. Also, the serum total bilirubin levels at baseline were marginally higher in patients with liver injuries than in those without liver injuries. The serum concentrations of DCV did not differ significantly between patients with and without liver injury, but the serum concentrations of ASV in patients with liver injuries tended to be higher than those in patient without liver injuries. Although the percentage of patients showing 3.25 or more of FIB4 index at baseline and 4 weeks did not differ, the percentage of patients showing 3.0 C.O.I or more of M2BPGi levels at 4 weeks after initiation of therapy was higher among patients with liver injuries than those without liver injuries. Multivariate logistic regression analysis, however, identified serum ASV concentration, but not the total bilirubin levels and M2BPGi levels at 4 weeks, as being factors significantly associated with liver injuries, with an odds ratio of 1.045 (95% confidence interval 1.011–1.082, p<0.05) (Table 2).

**Table 2:** Multivariate logistic regression analysis to identify factors associated with liver injuries in patients with genotype 1b HCV receiving daclatasvir plus asunaprevir therapy.

Factors	Odds ratio	95% CI	*p*-value
Albumin at 4 weeks (every 1.0 g/dL)	―	―	N.S.
Bilirubin at baselilne (every 1.0 mg/dL)	―	―	N.S.
M2BPGi level at 4 weeks ≧3.0 C.O.I	―	―	N.S.
ASV level (every 100 ng/dL)	1.045	1.011–1.082	0.010

## Discussion

Drug-induced liver injury (DILI) is an important adverse event developing during antiviral therapy for HCV, especially during treatment with DAAs including NS3/4A protease inhibitors. Previously, we reported that DILI during DCV plus ASV therapy could be classified into 2 types: immunoallergic and toxic liver injury [11, 12]. Immunoallergic liver injury mainly develops within 8 weeks after the initiation of DCV plus ASV therapy and is associated with eosinophilia and serum CRP elevation, while toxic liver injury occurs later than 8 weeks after the start of therapy [12]. Although we experienced one patient manifesting immune allergic liver injury at 3 weeks of DCV plus ASV therapy, the therapy was discontinued within 4 weeks in this patient. Thus, in the present study, patients manifesting immune allergic liver injuries were excluded from the cohort, and serum concentrations of DCV and ASV were measured during DCV plus ASV therapy in an attempt to clarify the mechanism underlying toxic liver injury, and found that the serum ASV concentrations at 4 weeks after the initiation of therapy were significantly and independently associated with liver injuries including elevation of serum AST and/or ALT levels to 150 U/L or more. Akuta, *et al*. previously reported that serum albumin levels affected serum ASV concentrations, and serum ASV concentrations might partly be contribute to provoke severe elevation of serum ALT levels [13]. The present study revealed that serum ASV concentrations were increased in correlation with the extent of liver fibrosis estimated from laboratory data, and strongly supported the observations reported by Akuta, *et al*.

In the present study, the extent of liver fibrosis was estimated from peripheral platelet counts and serum albumin levels at baseline and FIB4 index and serum M2BPGi levels at 4 weeks after the initiation of DCV plus ASV therapy. FIB4 index is calculated based on 4 parameters; age, peripheral platelet counts and serum AST and ALT levels [7]. Moreover, serum M2BPGi levels were shown to decrease rapidly along with disappearance of HCV-RNA during antiviral therapies using DAAs, and the levels following DAA therapy are recognized as a useful marker to estimate the risk underlying hepatocarcinogenesis following SVR achievement [14–16]. Thus, serum M2BPGi levels as well as FIB4 index may reflect the extents of hepatitis activity as well as liver fibrosis in patients with HCV, and the extent of liver fibrosis was estimated from FIB4 index and serum M2BPGi levels at 4 weeks of the therapy, when serum AST and ALT levels decreased along with serum HCV-RNA disappearance in almost all patients during DCV plus ASV therapy. Consequently, serum ASV concentrations were higher in patients showing 3.0 C.O.I or more of serum M2BPGi levels at 4 weeks of the therapy than in those with the levels less than 3.0 C.O.I, and were also positively correlated with the levels. Moreover, serum ASV concentrations showed positive correlation with FIB4 index at 4 weeks of the therapy and were correlated negatively with peripheral platelet counts and serum albumin level at baseline. Our observations were confirmed by those in the previous report by Morio *et al*. [17], in which serum ASV concentrations were shown to be higher in cirrhotic patients than in non-cirrhotic patients during DCV plus ASV therapy.

Eley, *et al*. reported that DAA therapies including ASV were not recommended to be done in patients with moderate-to-severe hepatic impairment [18]. In the present study, the univariate analysis revealed that serum albumin levels at 4 weeks were significantly lower among patients with liver injuries than those without liver injuries. Similar difference was seen in a percentage of patients manifesting 3.0 C.O.I or more of serum M2BPGi levels at 4 weeks of the therapies. Also, serum total bilirubin levels at baseline were marginally higher in patients with liver injuries than in those without liver injuries. Thus, careful observations should be done in patients with especially liver cirrhosis during DAA therapies including ASV. In a real-world clinical practice in Japan, however, doses of ASV were reduced in patients showing liver injuries during DCV plus ASV therapy, based on expert experience. In recent years, however, various DAA regimens have been approved. Thus, DAA therapies without NS3/4A protease inhibitors such as ledipasvir, an NS5A inhibitor, plus sofosbuvir, a nucleotide type NS5B polymerase inhibitor are recommended to be done in patients with extended liver fibrosis [19].

Serum M2BPGi levels were reported to be increased in association with serum transaminase levels [20]. Moreover, serum M2BPGi levels were also increased in patient with acute liver injury [21]. In the present study, serum M2BPGi levels at 4 weeks showed weak correlations with serum AST and ALT levels at 4 weeks (the correlation coefficients of 0.070 and 0.108, respectively). Furthermore, liver injuries developed later than 4 weeks of the therapies in most of the patients (median at 10 weeks, range 4 to 20 weeks). Thus, serum M2BPGi levels as well as FIB4 index at 4 weeks were not affected by liver injuries during DCV plus ASV therapy.

The safety and efficacy of DCV plus ASV therapy for genotype 1b HCV-infected patients with renal dysfunction and those receiving hemodialysis have been reported [22, 23, 24]. In the present cohort, serum creatinine levels and eGFR at both baseline and 4weeks did not associate with development of liver injuries. Furthermore, those values did not affect serum ASV and DCV concentrations because both agents are mainly metabolized in hepatocytes. These observations suggested that DCV plus ASV therapy is highly tolerant for patients with renal impairment.

Almost all approved DAAs, including ASV, have been shown to be substrates of organic anion transporting polypeptide (OATP) 1B1 and 1B3, which inhibit OATP1B1 function, with consequent development of liver injury caused by interaction with other substrate drugs of OATP1B1, such as HMG-CoA reductase inhibitors (statins) and angiotensin II receptor blockers (ARBs) [25]. In the present study, 17 (50.0%) of the 34 patients manifesting evidence of liver injury during DCV plus ASV therapy had received other drugs that could serve as substrates for OATP1B1. In these patients, increased serum levels of these concomitantly administered medications could have contributed to liver injuries. Also, it has been reported that ASV were metabolized by CYP3A4 and P-glycoprotein [26, 27], and that hepatic mRNA expressions of CYP3A4 and OATP1B1 decrease with the progression of liver fibrosis [28]. Therefore, serum ASV concentrations might increase due to the reduction of metabolic capacity for ASV associated with progression of liver fibrosis. Maekawa *et al*. reported that single-nucleotide polymorphism (SNP) of UGT1A1 (rs4148323) was associated with elevation of the serum AST and/or ALT levels during DCV plus ASV therapy in Japanese patients [29]. Thus, the differences in the serum ASV concentrations depending on the severity of liver fibrosis should be evaluated in the future in reference to UGT1A1 SNPs and hepatic mRNA expressions of CYP3A4 and OATP1B1.

In conclusion, high serum ASV concentrations were identified to be significantly associated with liver injuries during DCV plus ASV therapy in patients with genotype 1b HCV. Considering that serum ASV concentrations were correlated with laboratory parameters including serum M2BPGi levels at 4 weeks of the therapy which may reflect the extent of liver fibrosis, serum AST and ALT levels and liver function must be carefully monitored, especially in patients with progressive liver fibrosis.

## Supporting information

S1 File (PDF)

S2 File (PDF)

S3 File (PDF)
