# Supplementary material for: Serum asunaprevir concentrations showing correlation with the extent of liver fibrosis as a factor inducing liver injuries in patients with genotype-1b hepatitis C virus receiving daclatasvir plus asunaprevir therapy
Source: PLoS One. 2018 Oct 11;13(10):e0205600. doi: 10.1371/journal.pone.0205600 (PMC6181393; doi:10.1371/journal.pone.0205600)
Supplement: S1 File — (PDF) [file pone.0205600.s001.pdf]

UMIN-CTR Clinical Trial

Name:	UMIN ID:
Recruitment status	No longer recruiting
Unique ID issued by UMIN	UMIN000018857
Receipt No.	R000021811
Official scientific title of the study	Assessment of variants associated with resistance in NS3, NS5A and NS5B region in genotype 1b HCV infected patients
Date of disclosure of the study information	2015/10/01
Last modified on	2018/03/03

* This page includes information on clinical trials registered in UMIN clinical trial registed system.
* We don't aim to advertise certain products or treatments

Basic information	
Official scientific title of the study	Assessment of variants associated with resistance in NS3, NS5A and NS5B region in genotype 1b HCV infected patients
Title of the study (Brief title)	Assessment of RAVs
Region	Japan

Condition	
Condition	patients infected with genotype 1 HCV
Classification by specialty	Hepato-biliary-pancreatic medicine
Classification by malignancy	Others
Genomic information	NO

Objectives	
Narrative objectives1	To improve the therapeutic effects of antiviral therapy in chronic hepatitis C genotype 1 by evaluated the kinetics of RAVs in NS3, NS5A and NS5B region of HCV.
Basic objectives2	Efficacy
Basic objectives -Others	
Trial characteristics_1	
Trial characteristics_2	
Developmental phase	

Assessment	
Primary outcomes	the efficacy of antiviral therapy at post-treatment 12 and 24 weeks
Key secondary outcomes	

Base	
Study type	Observational

Study design	
Basic design	
Randomization	
Randomization unit	
Blinding	
Control	
Stratification	
Dynamic allocation	
Institution consideration	
Blocking	
Concealment	

Intervention	
No. of arms	
Purpose of intervention	
Type of intervention	
Interventions/Control_1	
Interventions/Control_2	
Interventions/Control_3	

Interventions/Control_4	
Interventions/Control_5	
Interventions/Control_6	
Interventions/Control_7	
Interventions/Control_8	
Interventions/Control_9	
Interventions/Control_10	

Eligibility	
Age-lower limit	Not applicable
Age-upper limit	Not applicable
Gender	Male and Female
Key inclusion criteria	HCV genotype 1 infected patients who recieve anti-viral therapy such as combination therapy with daclatasvis/asunaprevir, sofosbuvir/ledipasvir and ombitasvir/paritaprevir/ritonavir
Key exclusion criteria	patients infected with HCV other than genotype 1
Target sample size	300

Research contact person	
Name of lead principal investigator	Satochi Mochida
Organization	Saitama Medical University
Division name	Department of Gastroenterology & Hepatology
Address	38 Morohongo, Moroyama-Machi, Iruma-Gun, Saitama
TEL	049-276-1198
Email	smochida@saitama-med.ac.jp

Public contact	
Name of contact person	Yoshihito Uchida
Organization	Saitama Medical University
Division name	Department of Gastroenterology & Hepatology
Address	38 Morohongo, Moroyama-Machi, Iruma-Gun, Saitama
TEL	049-276-1198
Homepage URL	
Email	y_uchida@saitama-med.ac.jp

Sponsor	
Institute	Department of Gastroenterology & Hepatology,<br>Saitama Medical University
Institute	
Department	

Funding Source	
Organization	Japan agency for medical research and development (AMED)
Organization	
Division	
Category of Funding Organization	Japanese Governmental office
Nationality of Funding Organization	

Other related organizations	
Co-sponsor	
Name of secondary fun d er(s)	

Secondary IDs	
Secondary IDs	NO
Study ID_1	
Org. issuing International ID_1	
Study ID_2	
Org. issuing International ID_2	
IND to MHLW	

Institutions	
Institutions	

Other administrative information	
Date of disclosure of the study information	2015 Year 10 Month 01 Day

Progress	
Recruitment status	No longer recruiting
Date of protocol fixation	2015 Year 09 Month 01 Day
Anticipated trial start date	2015 Year 09 Month 14 Day
Last follow-up date	
Date of closure to data entry	
Date trial data considered complete	
Date analysis concluded	

Related information	
URL releasing protocol	
Publication of results	Unpublished
URL releasing results	
Results	
Other related information	We collect patients' serum samples at start and 1, 2 and 4 weeks of treatment and evaluate the variants associated with resistance in NS3, NS5A and NS5B region. When viral RNA are not negative at 4 weeks of treatment, we continue the serum sampling and evaluation every 2 weeks. In the cases that viral RNA are negative at 4 weeks of treatment, sera are collected when viral RNA are positive during anti viral therapy.

Management information	
Registered date	2015 Year 08 Month 30 Day
Last modified on	2018 Year 03 Month 03 Day

Link to view the page	
URL(English)	https://upload.umin.ac.jp/cgi-open-bin/ctr_e/ctr_view.cgi?recptno=R000021811

Research Plan	
Registered date	File name

Research case data specifications	
Registered date	File name

Research case data	
Registered date	File name

Back

Contact us.

UMIN Infrastructure for Academic Activities
University hospital Medical Information Network
