# Supplementary material for: Serum asunaprevir concentrations showing correlation with the extent of liver fibrosis as a factor inducing liver injuries in patients with genotype-1b hepatitis C virus receiving daclatasvir plus asunaprevir therapy
Source: PLoS One. 2018 Oct 11;13(10):e0205600. doi: 10.1371/journal.pone.0205600 (PMC6181393; doi:10.1371/journal.pone.0205600)
Supplement: S2 File — (PDF) [file pone.0205600.s002.pdf]

Study title: Mechanisms Involved in Development of Liver Injuries during Dual Oral Therapy with Daclatasvir Plus Asunaprevir in Patients with HCV Infection: The Significance of Serum Asunaprevir Concentrations.

Investigators: Prof. Satoshi MOCHIDA M.D., PhD.
Sub-investigators: Nobuaki NAKAYAMA M.D., PhD.
Yukinori IMAI M.D., PhD.
Mie INAO M.D., PhD.
Kayoko SUGAWARA M.D., PhD.
Yoshihito UCHIDA M.D., PhD.
Institution: Department of Gastroenterology & Hepatology, Saitama Medical University
Address: 38 Morohongo, Moroyama-cho, Iruma-gun, 350-0495 Saitama, Japan.
Tel & Fax: +81(0)49-276-1198
E-mail: smochida@saitama-med.ac.jp

Background

The resistance associated variants (RAVs) testing makes it possible to exclude patients with pre-existing RAVs for dual oral therapy (DUAL therapy) with daclatasvir (DCV) and asunaprevir (ASV). As a result, DUAL therapy is now comparable in efficacy to the competitors' regimens. On the other hand, management of adverse events, especially live injuries, remains an issue. Discontinuation of DUAL therapy is the only way to manage severe ALT elevation in the current labeling. It is assumed that ASV, but not DCV, may be responsible for adverse events especially for liver injuries, since abnormal liver tests frequently occurred during triple therapy with asunaprevir plus Peg-IFN and ribavirin [2], but was infrequent during therapy with daclatasvir, Peg-IFN and ribavirin [3]. Thus, in Japan, the ASV dose reduction is becoming common management of the ALT elevation. In our institute, more than 300 patients received DUAL therapy since September 2014. Increase of serum AST and/or ALT levels (>150 U/L) were observed in 25 patients (7.9%) and they received reduced dose of ASV [1]. However, no scientific data were available about safety/efficacy of DUAL regimen in patients who experienced dose reduction due to abnormal liver function value.

Aims：

To evaluate the effect of asunaprevir dose reduction on safety/effectiveness in the patients with abnormal liver function value during the DUAL therapy against non-reduced patients.

Hypothesis

ASV, but not DCV, may be responsible for adverse events especially for liver injuries, since abnormal liver tests frequently occurred during triple therapy with asunaprevir plus Peg-IFN and ribavirin [2], but was infrequent during therapy with daclatasvir, Peg-IFN and ribavirin [3].

Primary Endpoints/Objectives

To evaluate the effect of ASV dose reduction on safety/effectiveness in the patients with abnormal liver function value during the DUAL therapy against non-reduced patients.

Secondary Endpoints/Objectives

To evaluate the effect of ASV dose reduction on safety of DUAL therapy before and after reduction of ASV.

To evaluate the effect of cirrhosis on ASV dose reduction.

Exploratory Endpoints/Objectives

The relationship between the serum ASV or DCV concentrations and liver test were sequentially analyzed.

To evaluate the relationship between liver function and serum ASV or DCV concentrations during DUAL therapy.

To evaluate the relationship between therapeutic efficacy and serum ASV or DCV concentrations.

Study Design

Overview of Study Design

Blood samples were collected and conserved at baseline and at 7, 14, 28 days after initiation of DUAL therapy.

Trial Population
Japanese chronic hepatitis patients with HCV genotype 1
Age: 18 years or older
Gender: men and women

Inclusion Criteria
The patient with chronic genotype-1b HCV infection, who received DUAL oral therapy between September 2014 and today in Saitama Medical University Hospital were analyzed (retrospective study).

Exclusion Criteria
NA

Study Assessment
Main comparison will be done with 25 patients with the ASV dose reduction (R) and the matched patients without the dose reduction (NR). 1 or 2 baseline characteristics of subjects that a clinically important to find control subjects that match the cases, will be used.

Primary:
Efficay (SVR12), safety (Liver function; frequency and value) will be evaluated between R and NR.
Secondary:
Safety will be evaluated before and after the Dose reduction
Frequency of Dose reduction was compared between cirrhosis and non-cirrhosis groups.
Exploratory:
The demographic and clinical features, especially AST and ALT values for each concentrations of asunaprevir or daclatasvir, are summarized.

Treatment
All subjects enrolled in this study are treated with an approved regimen in Japan: DCV is administered orally at a dose of 60 mg once daily, and ASV is administered orally at a dose of 100 mg twice daily for 24 weeks. In case of increase of serum AST and/or ALT levels (>150 U/L) were observed, they received reduced dose of ASV

Sample size and sample size justification
Serum ASV and DCV concentrations were measured in 315 patients who received DUAL therapy. A total of 25 patients who were administered the reduced ASV dose, due to the increased AST/ALT levels (>150 U/L) during the therapies, and matching patients who did not experience the dose reduction..
We are planning a study with 25 experimental subjects and 50 control subjects for primary endpoint. In a previous study the response within each subject group was normally distributed with standard deviation 10. If the true difference in the experimental and control means is 10, we will be able to reject the null hypothesis that the population means of the experimental and control groups are equal with probability (power) .933. The Type I error probability associated with this test of this null hypothesis is 0.05..

Period of the Study
After IRB approval~31-Mar-2017

Data and Statistic Plan
Categorical data were compared using the Fisher's exact test. Distributions of continuous variables

were analyzed using the Student's *t*-test and/or the Mann-Whitney *U*-test. Logistic regression to determine the factors that differentiate cases from controls. All tests of significance were two-tailed, and *P* values of less than 0.05 were considered statistically significant.

Informed Consent

Written informed consent was obtained from all the patients prior to the collection of blood samples, which was conducted with the approval of the Institutional Review Board of Saitama Medical University Hospital.

Adverse Event Reporting

This study does not meet the criteria for adverse event reporting

References

[1] Fujii Y, Uchida Y, Mochida S. Reply to the letter entitled "Severe hepatotoxicity associated with asunaprevir and daclatasvir in chronic hepatitis C". ***Hepatology*** *2015 Aug 6. [Epub ahead of print].*

[2] Bronowicki JP, Pol S, Thuluvath PJ, Larrey D, Martorell CT, Rustgi VK, Morris DW, *et al.* Randomized study of asunaprevir plus pegylated interferon-α and ribavirin for previously untreated genotype 1 chronic hepatitis C. ***Antivir Ther*** *2013; 18: 885-893.*

[3] Suzuki F, Toyota J, Ikeda K, Chayama K, Mochida S, Hayashi N, Ishikawa H, *et al.* A randomized trial of daclatasvir with peginterferon alfa-2b and ribavirin for HCV genotype 1 infection. ***Antivir Ther*** *2014; 19: 491-499.*
