# Supplementary material for: Serum asunaprevir concentrations showing correlation with the extent of liver fibrosis as a factor inducing liver injuries in patients with genotype-1b hepatitis C virus receiving daclatasvir plus asunaprevir therapy
Source: PLoS One. 2018 Oct 11;13(10):e0205600. doi: 10.1371/journal.pone.0205600 (PMC6181393; doi:10.1371/journal.pone.0205600)
Supplement: S3 File — (PDF) [file pone.0205600.s003.pdf]

申請番号　16-053

ダクラタスビル・アスナプレビル併用療法における
薬物肝障害発生機序とアスナプレビル減量投与の
安全性と有用性に関する後方視的観察研究

研究計画書

＜研究責任者＞　　　　持田　智
＜研究機関名＞埼玉医科大学病院
＜所属＞　消化器内科・肝臓内科

１． 研究の目的

埼玉医科大学病院消化器内科・肝臓内科においてダクラタスビル（DCV）・アスナプレビル（ASV）併用療法を実施した genotype 1b の C 型慢性肝疾患患者 315 例を対象に，治療中に肝機能障害を生じ ASV を減量投与した症例の経過を後方視的に解析し, ASV の減量投与の安全性と有効性を検討する。

２． 研究の背景と意義

C 型肝炎ウイルス（HCV）の抗ウイルス療法は，2014 年 7 月に第 2 世代の NS3/4A プロテアーゼ阻害薬である ASV と NS5A 阻害薬である DCV が承認され，同薬剤を併用する経口のみの治療により 9 割以上の C 型慢性疾患患者においてウイルスの排除が可能となった。この経口 2 剤の治療は副作用が軽微であり，これまで IFN 併用療法を実施することが困難であった患者においても高い抗ウイルス効果を発揮する。しかし，IFN フリー経口 2 剤の治療においても副作用が出現することがある。同治療で，留意すべき主な副作用として薬物性肝障害が挙げられている。市販後に，当科で経口 2 剤療法を実施した 315 例のうち 25 例(8%)で肝機能障害を認め，grade-4 の肝機能障害で治療中止に至ったのは 9 例(3%)であった。このうちの 3 例は代償性肝硬変の症例で，いずれも治療開始前の child-pugh score が 6 点と肝予備能が低下している症例であった。

ASV，DCV ともに肝細胞で代謝される薬物であるが，これまでの検討から，ASV が用量依存性に肝機能障害を誘発すると考えられている。通常の診療では，経口 2 剤の治療中に肝機能障害を生じた症例は主治医の判断により ASV を減量することで，多くの症例は治療を完遂し，有効な治療効果を得られているが，同薬の減量投与に関してのエビデンスはない。本研究により，経口 2 剤の治療中に肝障害を生じた際に ASV を減量することの安全性と有用性を示すことは，同治療を中止せずに減量して治療を継続する指標となり，その社会的および医学的な意義は極めて大きい。

３．研究の実施体制・組織

１）主任研究者

所属：消化器内科・肝臓内科　　役職：教授　　　氏名：持田　智

２）研究実施者：別紙記載

３）共同研究機関及び研究責任者（多施設共同研究など、該当する場合）：該当なし

３）研究事務局及び担当者（該当する場合）：該当なし

４）上記以外のデータセンターなどの外部機関（該当する場合）：該当なし

４． 研究の方法と期間

１）デザイン

単施設後方視的観察臨床研究

２）研究対象者の登録基準/除外基準

埼玉医科大学病院消化器内科・肝臓内科において DCV・ASV 併用療法を実施した患者を対象とする。

３）目標症例数

全体症例数 315 例

埼玉医科大学病院における症例数 315 例

４）研究の期間

調査対象期間：2014 年 9 月 1 日～2016 年 3 月 31 日

研究期間：病院ＩＲＢ承認日～2017 年 3 月 31 日

５）スケジュール

病院 IRB 承認後より解析を開始する。

６）調査項目/検査項目

・治療経過中にアスナプレビルを減量した群（R 群）と減量しなかった群（NR 群）において，治療終了 12 週後のウイルス陰性化持続（SVR12）の達成頻度，有害事象の発生頻度を比較し，血中薬物濃度との関連性を評価する。

・肝硬変の有無別に，治療中のアスナプレビルの減量した症例の頻度，SVR12 の達成頻度，有害事象の発生頻度を比較し，血中薬物濃度との関連を評価する。

７）併用療法や併用薬剤の制限

後ろ向き観察研究のため該当なし。

８）中止基準

後ろ向き観察研究のため該当なし。

９）統計処理を行う方法

R 群 25 例に対して，年齢・性別・血液検査成績（Alb，AST，ALT，Bil，血小板数等）で傾向スコアマッチングさせた NR 群の 50 例（R 群 1 例に対して NR 群 2 例を割り付ける）を比較対象として，治療終了 12 週後のウイルス陰性化持続（SVR12）の達成頻度，有害事象の発生頻度の比較を行う。なお，R 群 25 例と NR 群の 50 例のサンプルサイズにおける検定力は 0.93，第 1 種過誤となる確率が 0.05 であり，妥当なサンプルサイズである。

１０）その他

なし

５． 研究に関する情報公開について

研究開始時のデータベースへの登録：非該当

データベースの名称：(　　)

登録番号：(　　　　　　　　　　　　　　)

６．試料・情報の保管について

本研究で得られた成績やその他の付帯情報は研究終了から 5 年，ないし研究結果の最終の公表から 3 年のどちらか遅い期日まで，埼玉医科大学消化器内科・肝臓内科の研究室にて保管する。その後，個人情報の漏洩に配慮し適切に廃棄する。また，本研究で得られた成績は将来の研究のために他の研究で使用される可能性があり，その際には改めて病院 IRB で承認を得た上で使用する。

７．個人情報保護の方法

当院単独の臨床研究であり、データの解析等もすべて当院で実施するため，被験者の氏名、ID、生年月日などの個人情報が外部に出ることはない。

８．インフォームドコンセント

後ろ向き研究のため、被験者からインフォームドコンセントは受けないが、研究の情報を公開し、研究対象者が拒否できる機会を保障する。

情報公開する場所

■埼玉医科大学病院ＩＲＢホームページ

URL: http://www.saitama-med.ac.jp/hospital/outline/irb.html

９．利益と不利益

本研究は後ろ向き観察研究であり，患者自身は直接的な利益，不利益を被ることはない。

１０．被験者に健康被害が生じた場合の補償内容

本研究は後ろ向き観察研究であり，患者自身に新たに健康被害が生じることはない。

１１．研究に関する被験者からの相談等の対応

以下の連絡先を相談窓口とする。

連絡先：埼玉医科大学病院　消化器内科・肝臓内科

昼間（9:00～17:00）：埼玉医科大学病院　消化器内科・肝臓内科外来　049-276-1279

夜間（17:00～9:00）：埼玉医科大学病院　消化器内科・肝臓内科病棟　049-276-1320

１２．費用に関する事項

１）研究の資金源

本研究はブリストル・マイヤーズ　スクイブ株式会社からの支援により実施される。

２）研究機関の研究に係る利益相反について

本研究に関わる利益相反は存在しない。

３）被験者への経済的負担

本研究では被験者への経済的負担は発生しない。

４）被験者への謝礼
本研究は被験者への謝礼はない。

１３．病院長への報告に関する事項

１）研究の実施の許可：
研究責任者は、研究の実施に先立ち、本研究計画書について病院ＩＲＢの承認及び病院長の許可を得ていることを確認する。
２）研究計画内容の変更：
研究責任者は、研究計画書内容や同意文書・説明文書内容に変更点が生じた場合は、速やかに病院長に変更申請をし、病院ＩＲＢの承認を得て、病院長の許可を得る。
３）実施状況報告：
研究責任者は、少なくとも年に１回以上の頻度で、研究の実施状況を病院長及び病院ＩＲＢに報告する。
４）研究終了時：
研究責任者は、研究が終了したら速やかに病院長と病院ＩＲＢに報告をする。
５）重篤な有害事象発現時の対応（侵襲ありの場合のみ）：
本研究は後ろ向き観察研究であり、新たな有害事象の発生はない。

１４．モニタリング・監査について（侵襲及び介入ありの場合）

該当せず。

１５．遺伝子検査に関する事項　（該当する場合のみ）

該当せず。

１６．研究結果の公表

日本肝臓学会，米国肝臓病会議，欧州肝臓病会議，アジア環太平洋肝臓会議など学術集会で発表するとともに，英文論文を作成して Hepatology Research，Hepatology，Journal of Hepatology，Hepatology International などの学会機関誌に発表する。

＜別紙＞　研究組織　一覧

１）研究実施者

氏名	所属（役職）
持田　智	消化器内科・肝臓内科（教授）
中山　伸朗	消化器内科・肝臓内科（准教授）
今井　幸紀	消化器内科・肝臓内科（准教授）
稲生　実枝	消化器内科・肝臓内科（准教授）
菅原　通子	消化器内科・肝臓内科（講師）
内田　義人	消化器内科・肝臓内科（助教）

２）共同研究機関及び研究責任者（該当なし）

施設名	診療科	研究責任医師

３）研究事務局及び担当者
該当なし。

４）上記以外のデータセンターなどの外部機関
該当なし。
